# Triad of Risk for Late Onset Alzheimer’s: Mitochondrial Haplotype, APOE Genotype and Chromosomal Sex

**DOI:** 10.3389/fnagi.2016.00232

**Published:** 2016-10-04

**Authors:** Yiwei Wang, Roberta D. Brinton

**Affiliations:** ^1^Department of Clinical Pharmacy, School of Pharmacy, University of Southern CaliforniaLos Angeles, CA, USA; ^2^Department of Pharmacology and Pharmaceutical Sciences, School of Pharmacy, University of Southern CaliforniaLos Angeles, CA, USA

**Keywords:** mitochondria, haplogroup, Alzheimer’s disease, APOE, sex

## Abstract

Brain is the most energetically demanding organ of the body, and is thus vulnerable to even modest decline in ATP generation. Multiple neurodegenerative diseases are associated with decline in mitochondrial function, e.g., Alzheimer’s, Parkinson’s, multiple sclerosis and multiple neuropathies. Genetic variances in the mitochondrial genome can modify bioenergetic and respiratory phenotypes, at both the cellular and system biology levels. Mitochondrial haplotype can be a key driver of mitochondrial efficiency. Herein, we focus on the association between mitochondrial haplotype and risk of late onset Alzheimer’s disease (LOAD). Evidence for the association of mitochondrial genetic variances/haplotypes and the risk of developing LOAD are explored and discussed. Further, we provide a conceptual framework that suggests an interaction between mitochondrial haplotypes and two demonstrated risk factors for Alzheimer’s disease (AD), apolipoprotein E (APOE) genotype and chromosomal sex. We posit herein that mitochondrial haplotype, and hence respiratory capacity, plays a key role in determining risk of LOAD and other age-associated neurodegenerative diseases. Further, therapeutic design and targeting that involve mitochondrial haplotype would advance precision medicine for AD and other age related neurodegenerative diseases.

## Introduction

**Figure 1:**
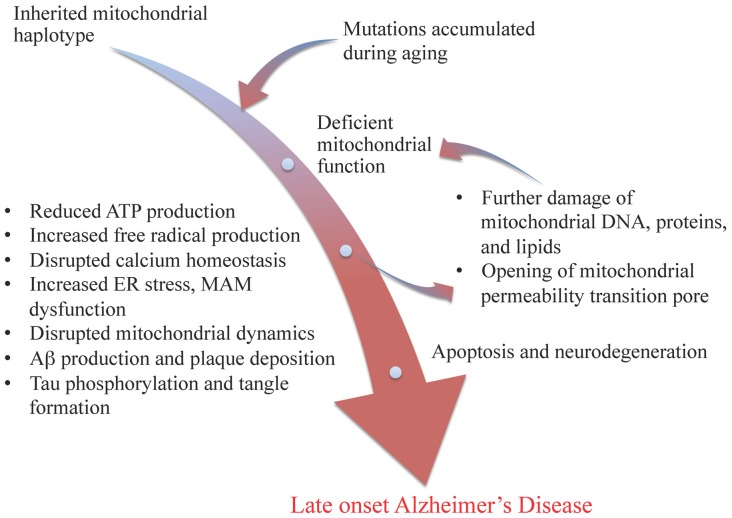
**Modified mitochondrial cascade of late-onset Alzheimer’s disease (LOAD).** Inherited mitochondrial genetic variations and accumulated mutations during aging lead to deficiency in mitochondrial functions, initiating a cascade of events including reduced ATP production, increased free radical formation and ER stress, disrupted mitochondrial associated membrane (MAM) function and mitochondrial dynamics, as well as amyloid beta plaque and tau tangle formation, which result in further damages of mitochondria and ultimately leads to apoptosis and neurodegeneration, such as LOAD.

A central role of mitochondria in age-related metabolic and neurodegenerative diseases has long been proposed, and an association between mitochondrial dysfunction and Alzheimer’s disease has been long proposed across multiple investigative strategies from analysis of human tissue to cell model systems (Beal, 1996; Gibson et al., 2000; Trimmer et al., 2000; Swerdlow and Khan, 2004; Bubber et al., 2005; Wallace, 2005; Lin and Beal, 2006; Brinton, 2008, 2009; Khusnutdinova et al., 2008; Simpkins et al., 2008; Coskun et al., 2012; Brinton et al., 2015). The “mitochondrial cascade hypothesis”, originally proposed by Swerdlow and Khan (2004) as an explanation for late onset Alzheimer’s disease (LOAD), proposed that inherited mitochondrial genetic variations and mutations accumulated from aging should occupy the apex of the disease cascade. Briefly, this hypothesis maintained that mitochondrial genetic variations and mutations lead to deficient electron transport chain function, resulting in less ATP production, increased free radical production, disrupted calcium homeostasis, beta amyloid production and plaque deposition, and tau phosphorylation and tangle formation. These results in turn lead to further damage of mitochondrial DNA, proteins, and lipids, and the opening of mitochondrial permeability transition pore, which ultimately leads to cell death and neurodegeneration (see Figure 1). Recent studies also indicated disrupted crosstalk between mitochondria and endoplasmic reticulum (ER) via mitochondrial associated membrane (MAM), as well as abnormal mitochondrial dynamics in the etiology of Alzheimer’s disease (AD; Trimmer et al., 2000; Baloyannis et al., 2004; Paillusson et al., 2013; Burté et al., 2015; Zhang et al., 2016; see Figure 1).

Here we review the link between mitochondrial dysfunction and the risk of AD, the contribution of mitochondrial genetic variances to the risk, and how the risk is modulated by other factors, such as apolipoprotein E (APOE) genotype and sex differences.

## Mitochondrial Genome and Haplotypes

**Figure 2:**
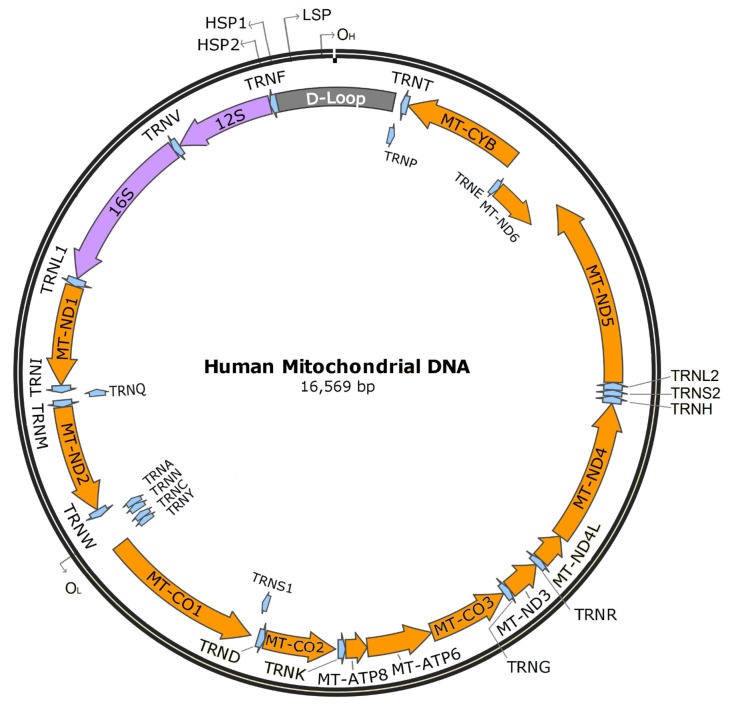
**Human mitochondrial DNA.** Orange indicates protein-encoding genes (13), purple indicates mitochondrial rRNAs (2), blue indicates tRNAs (22), and gray indicates the D-loop. O_H:_ heavy strand origin, O_L_: light strand origin, HSP1: major heavy strand promoter, HSP2: minor heavy strand promoter, LSP, light strand promoter.

Unlike many other organelles, mitochondria have their own genome. The human mitochondrial genome is a circular set of 16,569 base pairs encoding 37 genes. Thirteen of these genes encode protein subunits required for four of the five electron transport chain complexes: complex I (NADH ubiquinone oxidoreductase), complex III (cytochrome bc1 complex), complex IV (cytochrome c oxidase), and complex V (ATP synthase); two encode rRNAs for mitochondrial ribosomes (12S and 16S), and 22 encode tRNAs (see Figure 2).

Mitochondrial retention of their own genome throughout evolution solves two cell biology problems. First, the 13 electron transport subunits coded by mitochondrial DNA solves the problem that if they were generated by nuclear DNA, they would not cross the inner mitochondria membrane due to their high hydrophobicity (Popot and de Vitry, 1990). Second, the eukaryotic mitochondrial genome is transcribed and translated quite differently than the nuclear genome (Mercer et al., 2011). The genetic system of the mitochondria is transcribed as precursor polycistronic transcripts that are subsequently cleaved to generate mRNAs, tRNAs and rRNAs (Mercer et al., 2011). The mRNAs devoted to generating the 13 catalytic subunits required for oxidative phosphorylation are further translated using several non-universal codons unique to the mitochondrial translation machinery (Watanabe, 2010; Watanabe and Yokobori, 2011).

Like nuclear DNA, mitochondrial DNA undergoes mutation, though at a much higher rate (Miyata et al., 1982; Wallace et al., 1987), likely due to higher replication rate, a more mutagenic environment, and less efficient DNA repair (Xu et al., 2012). Unlike the nucleus, the mitochondrial DNA repair mechanism is largely limited to base excision repair. Mismatch as a result of either recombination or repair can lead to single nucleotide polymorphisms (SNPs).

Clusters of specific SNPs in the mitochondrial genome define mitochondrial haplogroups that reflect maternal lineage (Giles et al., 1980; Torroni et al., 1992). For example, the four lineages specific for sub-Saharan Africa are L0, L1, L2 and L3, and haplogroups A, B, C, D, G and F are common in Asia (Stewart and Chinnery, 2015). The major haplogroups within descendants of European ancestry are haplogroups H, I, J, K, M, T, U, V, W and X (Torroni et al., 1996). These haplogroups can be further classified into subhaplogroups or clustered together into superhaplogroups, such as superhaplogroup HV, JT, UK.

## Mitochondrial Dysfunction and Increased Risk of Alzheimer’S Disease

AD is a systemic disease with multiple etiologies (Morris et al., 2014). Early evidence linking mitochondrial dysfunction to AD dates back to a 1987 study by Sims et al. (1987), who found a reduced rate of oxygen uptake in the presence and absence of ADP in frontal neocortex of postmortem confirmed AD cases, indicating potential mitochondrial uncoupling in AD patients (Sims et al., 1987).

More direct evidence for the association between mitochondrial dysfunction and AD came later from studies on the activity of electron transport chain enzymes in AD patients and post mortem AD brain tissues. In the early 1990’s, reduced cytochrome c oxidase (complex IV) activity was observed in both platelets and postmortem AD brain (Parker et al., 1990, 1994). The reduction of cytochrome c activity in AD brain was later refined to the temporal cortex and hippocampus (Maurer et al., 2000). Supporting these findings, reduced mRNA levels of cytochrome c oxidase subunit 1 and 3 were observed in AD mid temporal gyrus and cytochrome c oxidase subunit 2 in AD hippocampus (Chandrasekaran et al., 1994; Aksenov et al., 1999). Furthermore, in both AD temporal and parietal cortices, protein levels of cytochrome c oxidase subunits were reduced, especially those encoded by mitochondria DNA (Kish et al., 1999). Although no reduction of cytochrome c oxidase content was observed in platelets of AD patients, cytoplasmic hybrids (cybrids) containing exogenous mitochondria extracted from platelets of AD patients showed less cytochrome c oxidase activity compared to cybrids harboring mitochondria from age-matched controls (Davis et al., 1997; Sheehan et al., 1997; Cardoso et al., 2004). Alternations in gene expression, protein level, and activity of other electron transport chain complexes have also been reported in AD tissues, though the evidence was less compelling and sometimes contradicted (Schagger and Ohm, 1995; Chandrasekaran et al., 1997; Aksenov et al., 1999; Kim et al., 2000; Bosetti et al., 2002; Bubber et al., 2005; Valla et al., 2006).

Upstream to electron transport chain, impairment of the tricarboxylic acid (TCA) cycle enzymes was observed in AD brain. Autopsy-confirmed AD brain had significantly reduced activity in pyruvate dehydrogenase complex, isocitrate dehydrogenase, and α-ketoglutarate dehydrogenase complex, whereas the activity of succinate dehydrogenase and malate dehydrogenase were increased (Bubber et al., 2005). The severity of enzyme activity impairment was also correlated with the clinical severity of Alzheimer’s pathology (Bubber et al., 2005). Decreased mitochondrial respiratory capacity was consistent with the higher level of lactate and lower key substrates for TCA cycle in cerebrospinal fluid and blood in AD patients (Redjems-Bennani et al., 1998; Mancuso et al., 2003).

Consistent with deficits in mitochondrial respiratory capacity, AD brain had elevated levels of peroxidation products in the frontal cortex, as well as decreased levels of superoxide dismutase, a radical defensive enzyme, in the frontal cortex, hippocampus, and cerebellum (Richardson, 1993). Increased oxidative stress is consistent with free radical damage of mitochondrial components and loss of mitochondrial membrane potential as observed in cybrids harboring mitochondria with AD origin (Cassarino et al., 1998; Trimmer et al., 2000).

Besides altered bioenergetic capacity, the crosstalk between mitochondria and the ER via MAM, which regulates many key functions of mitochondria, such as calcium uptake, phospholipid exchange, intracellular trafficking, ER stress and mitochondrial biogenesis, was also disrupted in AD (Paillusson et al., 2013; Burté et al., 2015).

Beyond changes in mitochondrial function, distribution and morphology of mitochondria were also different in AD patients. Neurons from AD brain harbored mitochondria of smaller sizes and disrupted mitochondrial cristae morphology, and cybrids created using mitochondria from sporadic AD patients also showed swollen mitochondria and less cristae (Trimmer et al., 2000; Baloyannis et al., 2004). The fission and fusion cycle of mitochondria also seemed disrupted in the hippocampus of AD brain (Zhang et al., 2016). The number of mitochondria was also significantly reduced, likely as a result of increased mitochondria degradation, turnover and autophagy (Hirai et al., 2001).

AD has a pattern of maternal inheritance, where inheritance of AD from mothers are more frequent than from fathers (Duara et al., 1993). The observation of maternal pattern of Alzheimer’s inheritance has been replicated over many decades by multiple groups, and has been linked to deficits in mitochondrial respiration and glucose metabolism apparently early in the aging process (Edland et al., 1996; Mosconi et al., 2011; Liu et al., 2013). In cognitively normal individuals, those with a maternal history of LOAD showed decline in platelet cytochrome c oxidase activity, compared to those with a paternal or no family history of the disease (Edland et al., 1996; Mosconi et al., 2011). In this same cohort, Mosconi et al. (2011) observed significantly lower brain glucose metabolism in persons with a maternal history of Alzheimer’s relative to those with a paternal or no family history of the disease. These data coupled with maternal inheritance of the mitochondrial genome strongly support a role for mitochondrial genetic variances in the etiology of the disease.

## Mitochondrial Haplotype is Associated with Different Respiratory, Metabolic and Bioenergetic Phenotypes

Mitochondrial genetic background can also affect metabolism and bioenergetic function. Resting metabolic rate (RMR) and total energy expenditure (TEE) were measured in 294 participants in the health, aging and body composition study (Health ABC; Tranah et al., 2011), including participants of cluster L, which contains common African haplogroups, and cluster N, which contains common European haplogroups (Tranah et al., 2011). Compared to N, cluster L had significantly lower RMR and TEE. Specifically, haplogroups L0, L2 and L3 had significantly lower RMR than haplogroup H and superhaplogroups UK and JT; haplogroup L3 had significantly lower TEE than haplogroup H and superhaplogroups UK and JT; haplogroup L2 had significantly lower TEE than haplogroup H and superhaplogroup JT (Tranah et al., 2011; see Table 1). In a cohort of healthy Spanish males, haplogroup J participants had significantly lower maximum oxygen consumption (VO_2max_) than non-J participants (Marcuello et al., 2009). This difference was later confirmed in an independent cohort of healthy Spanish males, where haplogroup H was determined to be the driving force for the difference (Martínez-Redondo et al., 2010; see Table 1).

**Table 1:** **Mitochondrial haplogroups/superhaplogroups differentially associated with respiratory phenotypes**.

	Relatively high	Relatively low	Reference
RMR	H, UK, JT	L2, L3, L3	Tranah et al. (2011)
TEE	H, UK, JT	L0, L2	Tranah et al. (2011)
VO_2max_	H	J	Marcuello et al. (2009) and
			Martínez-Redondo et al. (2010)

*Individuals of haplogroup/superhaplogroups H, UK and JT had higher rest metabolism rate (RMR), total energy differences (TEE) and/or VO_2max_ compared to individuals of cluster L and haplogroup J*.

The underlying cellular mechanism of the observed differences across different mitochondrial haplogroups was primarily elucidated using trans-mitochondrial cytoplasmic hybrids, or cybrids, which controlled for the nuclear genetic background to reveal mitochondrial variances. An early cybrids study using cultured A539 human lung carcinoma cells harboring either mitochondria of haplogroup H or T failed to identify any differences in bioenergetics function (Amo et al., 2008). However, differences in bioenergetics and mitochondrial function were identified in multiple later studies using different cell lines and mitochondrial haplogroups. Cybrids constructed from osteosarcoma 143B rho0 cells and platelets from healthy Spanish donors of either haplogroup H or superhaplogroup UK were investigated for mtDNA content (Gómez-Durán et al., 2010). Cybrids harboring UK superhaplogroup were found to have lower mtDNA content, lower mt-rRNA level, reduced protein synthesis, and decreased cytochrome c oxidase amount (Gómez-Durán et al., 2010). UK cybrids also had lower mitochondrial inner membrane potential and higher mitochondrial uncoupling, indicating potentially lower respiratory capacity and reduced ATP production (Gómez-Durán et al., 2010). Similar results were obtained in a later study in middle-age Caucasian males, where OXPHOS capacity normalized to citrate synthase content was found to be reduced by 24% in subjects with haplogroup U background comparing to those with haplogroup H background (Larsen et al., 2014). In 2013, cybrids constructed from human retinal epithelial cell line ARPE-19 and either haplogroup H or J mitochondria showed reduced ATP production and glycolysis in J cybrids (Kenney et al., 2013). In accordance with the observed reduction in mitochondrial respiration, J cybrids also showed lower ROS production (Kenney et al., 2013). Haplogroup J cybrids with chondrocyte nuclear genetic background also demonstrated lower NO levels than non-J cybrids (Fernández-Moreno et al., 2011). Similarly, major Asian mitochondrial haplogroups are also differentially associated with bioenergetic function (Lin et al., 2012). These associations identified in the human are also evident in animal models ranging from *Drosophila* to mice such that different mitochondrial genetic background is associated with differences in respiratory and metabolic phenotypes, electron transport chain enzyme activities and mitochondrial functions (Pichaud et al., 2012; Scheffler et al., 2012; Latorre-Pellicer et al., 2016).

Although different nuclear genetic background employed in the above studies make it difficult to compare the results across studies, this growing body of evidence supports mitochondrial genetic variance/haplotype as a key factor in the observed differences in bioenergetics and metabolism.

## Mitochondrial Genetic Variances/Haplotypes and Haplogroups and the Risk of Alzheimer’S Disease

In an early effort to assess the contribution of mitochondrial DNA variances to pathologies of neurodegenerative diseases, researchers identified a non-synonymous SNP in tRNA^Gln^, mt4336C, that had an increased frequency in a Caucasian cohort of late onset Alzheimer’s and Parkinson disease patients (Shoffner et al., 1993). The contribution of mt4336C was later confirmed in a different North American Caucasian cohort of AD patients (Hutchin and Cortopassi, 1995). Individuals harboring the mt4336C SNP also tended to harbor the mt16304C SNP, and had a more closely related D-loop sequence, which could be traced back to a single phylogenetic node (Shoffner et al., 1993; Hutchin and Cortopassi, 1995). However, two other studies did not confirm either mt4336C or mt16304C as a risk factor for developing AD in similar populations, when blood samples and leukocytes from clinically diagnosed patients were used instead of histopathologically confirmed postmortem AD brain tissues (Wragg et al., 1995; Zsurka et al., 1998). Today, mt16304C is known as a defining SNP for subhaplogroup H5, and mt4336C a defining SNP for subhaplogroup H5a. The above studies constituted the earliest debate over whether mitochondrial genetic variances can modify the risk of developing AD.

In fact, haplogroup H and superhaplogroup HV, which contains haplogroup H and its subhaplogroups, have been the most reported haplogroup in association with increased risk of developing AD (Chinnery et al., 2000; Edland et al., 2002; van der Walt et al., 2004, 2005; Elson et al., 2006; Fesahat et al., 2007; Mancuso et al., 2007; Maruszak et al., 2009, 2011; Santoro et al., 2010; Coto et al., 2011; Ridge et al., 2012; Fachal et al., 2015; see Table 2). A study based on 30 Iranian late onset Alzheimer’s patients and 100 controls found that haplogroup H was significantly more abundant in the disease group (Fesahat et al., 2007; see Table 2). In a Spanish-Caucasian group, haplogroup H and its defining SNP mt7028C were enriched in LOAD patients compared to controls (Coto et al., 2011; see Table 2). In a large Caucasian cohort containing 422 late-onset AD patients and 318 neurologically healthy controls, researchers found that superhaplogroup HV, which contains haplogroup H, had significantly higher presence in LOAD than in control (Maruszak et al., 2011; see Table 2). Finally, a meta-analysis pooling data from five previous studies (some studies including early-onset AD patients) also confirmed the association between haplogroup H and superhaplogroup HV and the risk of developing AD (Maruszak et al., 2011; see Table 2).

**Table 2:** **Observed effects of mitochondrial superhaplogroup HV and haplogroup H on risk of Alzheimer’s disease (AD)**.

Haplogroups	Observations	Reference
HV	Increased risk, especially in females	Maruszak et al. (2011)
	No effect	Elson et al. (2006), Maruszak et al. (2009) and Fachal et al. (2015)
H	Increased risk	Fesahat et al. (2007), Maruszak et al. (2009, 2011) and Coto et al. (2011)
	Defining SNP mt7028C increased risk	Coto et al. (2011)
	Defining SNP mt7028C increased risk in females only	van der Walt et al. (2004)
	H5 increased risk, especially in females	Santoro et al. (2010)
	H5 and APOE4 synergistically increased risk	Maruszak et al. (2011)
	H5a defining SNP mt4336C increased risk in APOE4 carriers	Edland et al. (2002)
	H6a1a and H6a1b decreased risk	Ridge et al. (2012)
	No effect	Chinnery et al. (2000), van der Walt et al. (2005), Mancuso et al. (2007) and Fachal et al. (2015)

*Effects of haplogroup H defining single nucleotide polymorphisms (SNPs) and its subhaplogroups are listed under haplogroup H*.

When mitochondrial DNA was sequenced in greater depth, subhaplogroup H5 was significantly associated with AD compared to haplogroup H of central-northern Italians, (Santoro et al., 2010; see Table 2). However, from the Cache county study on aging and memory in Utah residents, subhaplogroups H6a1a and H6a1b were found to be protective against AD (Ridge et al., 2012; see Table 2). While the protective role of subhaplogroups H6a1a and H6a1b seems contradictory to the overall risk of haplogroup H, the data predict that the observed risks within haplogroup H may be driven by its sub-haplogroups with H5 increases risk of LOAD whereas H6 reduces risk.

The second most studied superhaplogroup is UK, and its member haplogroups U and K, including their subhaplogroups (While haplogroup K is currently recognized as a branch of haplogroup U, early studies classified haplogroups U and K as two parallel haplogroups under superhaplogroup UK. For consistency of referring to previous studies, we will use the earlier classification system throughout this review article; see Table 3). In a Utah based ADNI cohort, superhaplogroup UK was identified as a risk factor for AD (Lakatos et al., 2010; see Table 3). These findings are in contrast to an earlier study conducted in a Poland-based Caucasian population, where no effect of superhaplogroup UK was observed (Maruszak et al., 2009; see Table 3). The disparity may be explained by differences in the distribution of specific subhaplogroups or SNPs in the studied populations. Specifically, while each of the three defining SNPs for haplogroup U (mt11467G, mt12308G and mt12372A) has been identified as a risk factor for AD (Lakatos et al., 2010), subhaplogroup U5a1 and SNP mt16224C, a haplogroup K defining SNP, were shown to be protective (Maruszak et al., 2011; see Table 3).

**Table 3:** **Observed effects of mitochondrial superhaplogroup KU and haplogroups K and U on risk of AD**.

Haplogroups	Observations	Reference
UK	Increased risk	Lakatos et al. (2010)
	Decreased risk in males	Maruszak et al. (2011)
	No effect	Maruszak et al. (2009) and Fachal et al. (2015)
K	Defining SNP mt16224C decreased risk	Maruszak et al. (2011)
	Decreased risk in APOE4 carriers	Carrieri et al. (2001) and Maruszak et al. (2011)
	No effect	Chinnery et al. (2000), van der Walt et al. (2005), Elson et al. (2006), Fesahat et al. (2007), Mancuso et al. (2007) and Maruszak et al. (2009)
U	Increased risk	Fesahat et al. (2007)
	Increased risk in males	van der Walt et al. (2004)
	Defining SNPs mt11467G, mt12308G, and mt12372A individually increased risk	Lakatos et al. (2010)
	Decreased risk in females	van der Walt et al. (2004)
	Decreased risk in APOE4 carriers	Carrieri et al. (2001)
	U5a1 decreases risk	Maruszak et al. (2011)
	No effect	Chinnery et al. (2000), van der Walt et al. (2005), Elson et al. (2006), Mancuso et al. (2007), Maruszak et al. (2009) and Fachal et al. (2015)

Another common European haplogroup studied for its association with AD is haplogroup T, where one study in French-Canadians found that the frequency of SNPs mt709A and mt15928A, both defining SNPs for haplogroup T, were three times higher in controls than in AD patients, suggesting a protective role of haplogroup T (Chagnon et al., 1999; see Table 4). However, the Health, Aging, and Body Composition (Health ABC) study found that haplogroup T had increased risk for dementia when compared to haplogroup H (Tranah et al., 2012). The Health ABC study also found that haplogroup J, also under superhaplogroup JT, was associated with significant decline in cognitive function compared to haplogroup H (Tranah et al., 2012).

**Table 4:** **Observed effects of mitochondrial superhaplogroup JT and haplogroups J and T on risk of AD**.

Haplogroups	Observations	Reference
JT	Decreased risk in females	Maruszak et al. (2011)
	No effect	Maruszak et al. (2009) and Fachal et al. (2015)
J	Decline in cognitive function comparing to H	Tranah et al. (2012)
	J2b defining SNP mt7476T, mt5633T, and mt15812A increased risk	Chagnon et al. (1999)
	No effect	Chinnery et al. (2000), van der Walt et al. (2005), Elson et al. (2006), Fesahat et al. (2007), Mancuso et al. (2007); Maruszak et al. (2009) and Fachal et al. (2015)
T	Increased risk for dementia comparing to H	Tranah et al. (2012)
	T defining SNP mt709A and mt15928A decreased risk	Chagnon et al. (1999)
	Decreased risk in females	Maruszak et al. (2011)
	No effect	Chinnery et al. (2000), van der Walt et al. (2005), Elson et al. (2006), Fesahat et al. (2007), Mancuso et al. (2007), Maruszak et al. (2009) and Fachal et al. (2015)

In addition to major European haplogroups, several African and Asian haplogroups have also been reported to be associated with the AD or risk of dementia (see Table 5). For example, in an African American population, haplogroup L1 was found to have increased risk for developing dementia (Tranah et al., 2014). In Asians, subhaplogroups G2a, B4c1 and N9b1 were reported to be associated with AD in Japanese populations, and haplogroup B5 was reported to be associated with AD in Han Chinese (Takasaki, 2008, 2009; Bi et al., 2015; see Table 5).

**Table 5:** **Association between some Asian mitochondrial haplogroups and the risk of Alzheimer’s disease**.

Haplogroups	Observations	References
L1	Increased risk	Tranah et al. (2014)
G2a	Increased risk	Takasaki (2008, 2009)
B4c1	Increased risk	Takasaki (2009)
N9b1	Increased risk	Takasaki (2009)
B5	Increases risk	Bi et al. (2015)

*Studies listed in chronological order*.

As reviewed here, the field has not reached consensus on the effect of mitochondrial haplogroups on late onset AD (LOAD). Different sets of haplogroups are identified in different studies and different studies could identify opposite effects of the same mitochondrial haplotype on risk of LOAD. Moreover, multiple studies involving Caucasians of European descendant (European descents in UK and US, Tuscans, Spanish and Old order Amish from Indiana and Ohio) did not detect associations between any haplogroup and AD (Chinnery et al., 2000; van der Walt et al., 2005; Elson et al., 2006; Mancuso et al., 2007; Fachal et al., 2015; see Tables 2–5). While the controversy may be driven by differences in the distribution of subhaplogroups within the studied population or different DNA sources (e.g., postmortem brain tissue vs. peripheral blood), the discrepancies between studies also suggested that as intriguing as the results are, mitochondrial genetic variances are unlikely to be the sole driving force of LOAD. Further, the most consistent risk factor for LOAD is APOE genotype, but it alone is not an absolute determinant. Below, we determine the relationship between mitochondrial haplogroup and APOE genotype on risk of AD.

## The Interaction between Mitochondrial Haplogroup and APOE Genotype on Risk of AD

APOEε4 genotype is a widely recognized risk factor for AD, and has been repeatedly confirmed in the studies reviewed herein (Corder et al., 1993; Poirier et al., 1993; Rebeck et al., 1993; Saunders et al., 1993; Carrieri et al., 2001; Edland et al., 2002; Coto et al., 2011; Maruszak et al., 2011). Further, APOEε4 has been associated with mitochondrial dysfunction and glucose hypometabolism in brain (Reiman et al., 2001, 2004, 2005; Mosconi et al., 2004a,b,c, 2005, 2008; Valla et al., 2010; Wolf et al., 2013). Compared to non-carriers, APOEε4 carriers showed reduced cerebral parietal glucose metabolism among cognitive normal elderlies with family history of AD (Small et al., 1995, 2000). In APOEε4 positive MCI patients, reduced regional cerebral metabolic rate of glucose consumption (rCMRglc) was detected in temporoparietal and posterior cingulate cortex (Mosconi et al., 2004b). In AD patients, more severe hypometabolism was detected in the parietal, temporal, and cingulate areas in APOEε4 carriers than non-carriers (Mosconi et al., 2004c; Drzezga et al., 2005). Brain glucose hypometabolism was also more widespread in APOEε4 positive AD patients (Mosconi et al., 2004a). On the therapeutic side, mild-to-moderate AD patients who are APOEε4 carriers were shown to be less responsive to rosiglitazone, which can improve mitochondrial efficiency and glucose metabolism (Risner et al., 2006; Roses et al., 2007).

In longitudinal studies, APOEε4 carriers had significantly greater rCMRglc decline in the vicinity of temporal, posterior cingulate, and prefrontal cortex, basal forebrain, parahippocampal gyrus, and thalamus (Reiman et al., 2001; Mosconi et al., 2008). Decrease of glucose metabolism was also evident in young and middle-aged APOEε4 carriers in posterior cingulate, parietal, temporal, and prefrontal cortex, as well as thalamus (Reiman et al., 2004; Mosconi et al., 2008). The effect of APOEε4 allele has also been shown to be gene dose dependent with APOEε4 homozygote carriers showing greater hypometabolic deficit relative to APOEε3/4 heterozygote carriers (Reiman et al., 2005).

At the cellular level, APOEε4 gene expression in human was associated with down-regulation of genes involved in mitochondrial oxidative phosphorylation and energy metabolism (Xu et al., 2006, 2007). APOEε4 gene expression was also found to be associated with lower mitochondrial cytochrome oxidase activity in posterior cingulate cortex among young adults with family history of AD (Valla et al., 2010). Neurons from humanized APOEε4 knock-in mice had significantly lower amount of all five electron transport chain complexes comparing to those from APOEε3 knock-in mice (Chen et al., 2011). Proteomic analysis revealed decreased expression of proteins involved in the TCA cycle, glucose, lipid and amino acid metabolism in APOEε4 knock-in mice (Shi et al., 2014). Further, cytochrome c levels were significantly lower in ApoEε4 mice compared with ApoEε3 mice (Shi et al., 2014). *In vitro* studies also suggested that truncated APOEε4 fragment can interact directly with mitochondria and cause mitochondrial dysfunction and neurotoxicity (Chang et al., 2005; Mahley et al., 2007). Given the association between decreased bioenergetic capacity in brain and the risk of AD, an interaction between APOE genotype and mitochondrial haplotypes is possible.

After stratifying by APOEε4 status, three interesting modes of interactions between APOEε4 status and mitochondrial haplogroups in modulating the risk of AD were apparent (see Table 6).

**Table 6:** **Three modes of interactions between APOEε4 status and mitochondrial haplogroups in modulating the risk of AD**.

Interactions	Haplogroups/SNPs	Reference
Neutralizing	K	Carrieri et al. (2001) and
		Maruszak et al. (2011)
	U	Carrieri et al. (2001)
Enabling	mt4336C (H)	Edland et al. (2002)
Synergistic	H5	Maruszak et al. (2011)
	mt7028C (H)	Coto et al. (2011)

*Haplogroups K and U could neutralize the risk of APOEε4 on AD. Haplogroup H defining SNP mt4336C was associated with LOAD in APOEε4 carriers only. Subhaplogroup H5 and haplogroup H defining SNP mt7028C could act synergistically with APOEε4 to increase the risk of LOAD*.

The first mode is a neutralizing effect of mitochondrial haplogroup on the effect of APOEε4 on risk of AD (see Table 6). Early studies identified a non-random association between mitochondrial haplogroup and APOEε4 status in AD patients (Carrieri et al., 2001). Specifically, while APOEε4 carriers had significantly higher odds ratio for AD, those belonging to haplogroups K and U did not, indicating a neutralizing effect of haplogroups K and U on the risk of APOEε4 gene status (Carrieri et al., 2001). The non-random distribution of mitochondrial haplogroups associated with APOEε4 status and the neutralizing effect of mitochondrial haplogroup K on APOEε4 were later confirmed by Maruszak et al. (2011).

The second mode is an enabling effect of APOEε4 on mitochondrial genetic variances as risk factors for AD (see Table 6). In non-APOEε4 carriers, SNP mt4336C (a defining SNP for subhaplogroup H5a) was not an AD risk factor, however, in APOEε4 carriers, the same SNP was a risk factor for AD (Edland et al., 2002). This study indicated that APOE genotype could explain the earlier disparity regarding the association between SNP mt4336C and AD (Shoffner et al., 1993; Hutchin and Cortopassi, 1995; Wragg et al., 1995; Zsurka et al., 1998).

A synergistic effect was also observed between APOEε4 and mitochondrial haplotypes (see Table 6). For example, SNP mt7028C, a defining SNP for haplogroup H, and subhaplogroup H5 were suggested to act synergistically with APOEε4 to increase risk for AD (Coto et al., 2011; Maruszak et al., 2011).

As with the association between mitochondrial haplotype and the risk of late onset AD, the interaction between APOEε4 and mitochondrial genetic variances in modulating the risk of late onset AD remains debatable. Multiple studies failed to identify any correlation between mitochondrial haplogroup and APOEε4 status or failed to identify an interaction between the two on the risk of developing AD (Zsurka et al., 1998; Chinnery et al., 2000; van der Walt et al., 2005; Mancuso et al., 2007; Lakatos et al., 2010; Santoro et al., 2010; Ridge et al., 2013). Collectively, these disparate findings on the association between mitochondrial haplotype, APOE genotype, and risk of AD emphasize the importance of a precision medicine approach that considers mitochondrial genetic variance in combination with nuclear genetics.

## Mitochondrial Haplogroup and Sex Differences and Risk of Alzheimer’S Disease

Females are at greater lifetime risk for LOAD, and also have higher prevalence and incidence rate than all age-matched males (Brookmeyer et al., 1998; Mielke et al., 2014; Grimm et al., 2016). The higher risk for female is also evident in faster disease progression and greater burden of AD pathology (Aguero-Torres et al., 1998; Corder et al., 2004; Barnes et al., 2005; Skup et al., 2011; Kelly et al., 2013; Mielke et al., 2014; Grimm et al., 2016). While the underlying mechanism remains to be elucidated, increased mitochondrial oxidative stress may play a role (Schuessel et al., 2004; Mandal et al., 2012). Given the effect of mitochondrial genetic variances on mitochondrial function and risk of AD, it is of interest to identify any interaction between mitochondrial haplotypes and sex difference on risk of LOAD.

While almost all the reviewed studies recognized sex differences on risk of LOAD, only a few studied the effects of mitochondrial genetic variances in each sex (van der Walt et al., 2004; Maruszak et al., 2009, 2011; Santoro et al., 2010; see Table 7). Within these studies, some mitochondrial genetic variances were found to be associated with AD in females only (van der Walt et al., 2004; Maruszak et al., 2009, 2011; Santoro et al., 2010). Superhaplogroup JT, haplogroup T, a haplogroup T defining SNP mt13368A, a haplogroup U defining SNP 12308G and a non-H defining SNP mt7028T were found to exert protective effects only in females (van der Walt et al., 2004; Maruszak et al., 2011; see Table 7). Superhaplogroup HV, haplogroup H, subhaplogroup H5 and a haplogroup H defining SNP mt7028C were identified as risk factors for only in females (Maruszak et al., 2009, 2011; Santoro et al., 2010; see Table 7).

**Table 7:** **Sex differentiates effects of mitochondrial haplogroups on risk of AD**.

Haplogroups/SNPs	Female	Male	Authors
H	Increased risk	No effect	Maruszak et al. (2009, 2011)
H5	Increased risk	No effect	Santoro et al. (2010)
HV	Increased risk	No effect	Maruszak et al. (2009, 2011)
T	Decreased risk	No effect	Maruszak et al. (2011)
JT	Decreased risk	No effect	Maruszak et al. (2011)
U	Decreased risk	Increased risk	van der Walt et al. (2004)
KU	No effect	Decreased risk	Maruszak et al. (2011)
mt7028C (H)	Increased risk	No effect	Maruszak et al. (2009)
mt13368A (T)	Decreased risk	No effect	Maruszak et al. (2011)
mt13708G (non-J)	No effect	Increased risk	Maruszak et al. (2009)
mt9055A (K)	No effect	Decreased risk	Maruszak et al. (2011)

*Haplogroups and SNPs identified to have sex differences from literatures are listed. A possible haplogroup defined by the SNPs listed is indicated in the parenthesis following the SNP*.

In contrast, some variances affected only males (see Table 7). Superhaplogroup UK, and SNP mt9055A, a defining SNP for haplogroup K, were found to be associated with reduced risk of AD in males, while SNP mt13708G (for many non-J haplogroups), and SNP mt10398A, a defining SNP for some subhaplogroups of U, were associated with increased risk only in males (van der Walt et al., 2004; Maruszak et al., 2009, 2011; see Table 7). Certain mitochondrial genetic variances also showed opposite effects in each sex. For example, haplogroup U was associated with increased risk in males but decreased risk in females (van der Walt et al., 2004; see Table 7).

These results indicate that previously observed associations between mitochondrial haplogroups and AD could be driven by sex. Conversely, the effects observed in one sex could be diluted in the whole population, or be neutralized by the other sex, resulting in non-significant associations.

## Conclusion and Perspectives

Mounting evidence suggested a central role of mitochondrial dysfunction in the etiology of late onset AD. As reviewed above, disruption of mitochondrial bioenergetics, structure and dynamics have all been indicated in AD patients. Given the maternal pattern of inheritance of late onset AD and mitochondrial genome, herein we reviewed the association of mitochondrial genetic variances on bioenergetics, respiratory phenotypes and risk of developing LOAD. While the outcomes remain debatable, a large body of science supports an association between mitochondrial genetic variances and differences in bioenergetics and AD susceptibility. Several factors can help explain the disagreement. First, although most studies were conducted in descendants of European origin, the geographic distribution of participants were drastically different, which could result in diverse nuclear genetic background. Since mitochondria communicate extensively with the nucleus, uncontrolled nuclear background can potentially mask effects of mitochondrial genetic variances. Second, as we have reviewed, observations could be driven by certain subhaplogroups, thus results obtained from different populations may be biased by their dominant subhaplogroup. Third, given the heteroplasmic nature of the mitochondrial DNA, accumulated mutations throughout aging may be haplotype and tissue specific (Wallace, 2005), which could contribute to the discrepancies between studies that had different sources of mtDNA (e.g., brain tissues vs. peripheral blood samples; Wallace, 1994). And last, the criteria for mitochondrial haplogroup assignment evolved during the past two decades. Initial studies used haplotype assignment based on only 10 SNPs in the control region, whereas more recent studies used 138 SNPs across the whole human mitochondrial genome. As a result, some ambiguous assignments were resolved and more subhaplogroups were identified.

**Figure 3:**
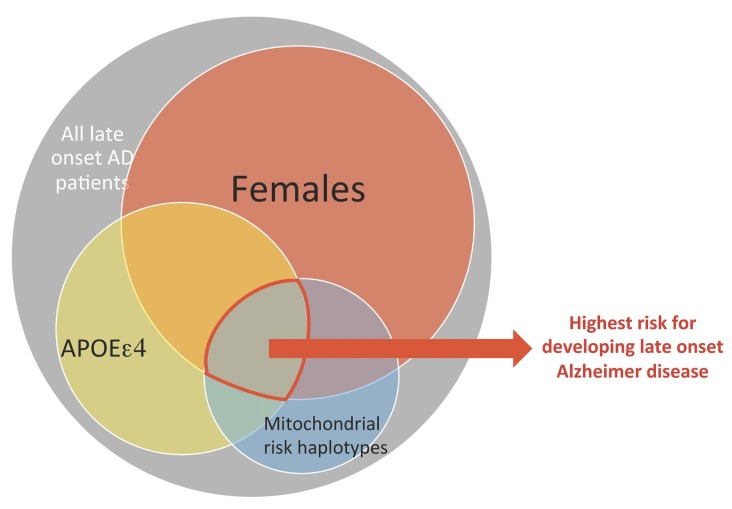
**A conceptual framework where the combination of mitochondrial and nuclear genetic risk factors plus sex as a higher risk factor for LOAD.** Each of the three factors: sex, APOEε4 genotype and mitochondrial genetic variance has moderate risk for developing late onset AD, but constitutes a much higher risk when combined.

The data also indicate that mitochondrial haplotype is one factor among several that impacts risk of AD. This is consistent with the multifactorial nature of aging trajectories and risk for LOAD. A systems biology approach that integrates mitochondrial genetic variances and risk factors such as APOEε4 genotype and sex is a step towards resolving disparities across studies of mitochondrial haplotype and risk of neurodegenerative diseases associated with mitochondrial dysfunction (see Figure 3). More importantly, we propose a precision medicine approach, where nuclear genetic risk factors (especially APOEε4 genotype), mitochondrial haplotypes, and sex differences can be incorporated into future therapeutic designs for LOAD.

## Author Contributions

YW and RDB wrote and reviewed the manuscript together.

## Conflict of Interest Statement

The authors declare that the research was conducted in the absence of any commercial or financial relationships that could be construed as a potential conflict of interest.
